# VOLCORE, a global database of visible tephra layers sampled by ocean drilling

**DOI:** 10.1038/s41597-020-00673-1

**Published:** 2020-10-06

**Authors:** Sue H. Mahony, Nicholas H. Barnard, R. Stephen J. Sparks, Jonathan C. Rougier

**Affiliations:** 1 School of Earth Sciences, Wills Memorial Building, University of Bristol, Queen’s Road, Bristol, BS8 1RJ United Kingdom; 2 School of Mathematics, University of Bristol, University Walk, Bristol, BS8 1TW United Kingdom

**Keywords:** Volcanology, Palaeoclimate, Sedimentology, Geodynamics, Palaeoceanography

## Abstract

The VOLCORE (Volcanic Core Records) database is a collection of 34,696 visible tephra (volcanic ash and lithological or grain size variations) occurrences reported in the initial reports volumes of all of the Deep Sea Drilling Project (DSDP; 1966–1983), the Ocean Drilling Program (ODP; 1983–2003), the Integrated Ocean Drilling Program (IODP; 2003–2013) and the International Ocean Discovery Program (IODP; 2013-present) up to and including IODP Expedition 381. The combined international ocean drilling programmes (OD) have locations with global coverage. Cored tephra layers and tephra-bearing sediments span timescales from recent to ~150 million years in age. This database is a collection of information about reported visible tephra layers entirely or predominantly composed of volcanic ash. Data include the depth below sea floor, tephra thickness, location, and any reported comments. An approximate age was estimated for most (29,493) of the tephra layers using published age-depth models. The database can be used as a starting point for studies of tephrochronology, volcanology, geochemistry, studies of sediment transport and palaeoclimatology.

## Abstract (summary)

**Table:** 

Measurement(s)	tephra
Technology Type(s)	digital curation
Factor Type(s)	age • geographic location
Sample Characteristic - Environment	ocean floor
Sample Characteristic - Location	global

Machine-accessible metadata file describing the reported data: 10.6084/m9.figshare.12951347

## Background & Summary

An important contribution of ocean drilling is to provide records of explosive volcanism through the occurrences of visible volcanic tephra (volcanic ash and lithological or grain size variations) layers and volcanic tephra dispersed in sediment (cryptotephra). This paper documents and describes a comprehensive global database of visible tephra layers identified in cores from the DSDP, ODP and IODP programs. For brevity we refer to the different programmes collectively as OD (Ocean Drilling). The database is called VOLCORE (Volcanic Core Records). The VOLCORE database collates every record of tephra from the OD archive of the Visual Core Description (VCD) forms (produced during the initial description of the sediments). VOLCORE only records visible tephra, not cryptotephra, which are invisible to the naked eye. Cryptotephra are an important record of volcanism, but are not included in VOLCORE because this information is not routinely available in the VCD archive. Tephra deposits reported in VOLCORE predominantly represent a record of large magnitude explosive volcanism^1^ but may include deposits formed by other processes, e.g. flank collapse deposits, turbidites or hyaloclastites from submarine volcanism.

The occurrence and preservation of a visible tephra layer in a core is affected by many factors. Occurrence depends on wind direction and distance from the source volcano. Eruption magnitude is a major factor and investigations of OD cores around Japan indicates that visible tephra layers are predominantly a record of large magnitude explosive eruptions^1^. Preservation is dependent on factors, such as reworking by ocean currents, bioturbation and topographic effects leading to local redistribution of tephra. Faulting may remove or duplicate layers. Disturbance of layers can occur during drilling. Shipboard identification varies between core describers and errors may occur during final data collection. These issues are explored further in the Validation and Usage Notes sections of this report.

Recovering volcanic records has been a major objective of some ocean drilling legs (e.g. IODP Expedition 340, ODP Expeditions 165 or 157), but more commonly the records are a by-product of expeditions focussed on other geoscience questions^2^. Visible tephra layers can provide significant stratigraphic horizons, can be correlated between cores and provide opportunities for precise dating to support the development of accurate age-depth models for sediment sequences in cores. Studies of tephra layers allow progress to be made in understanding regional volcanic histories, evolution of volcanic islands, and links between volcanism, tectonics and climate. The visible tephra record can constrain rates of volcanism and shed light on transport processes in the atmosphere and oceans. Tephra layers also provide the potential to investigate explosive volcanism on a global scale, but there have been few global-scale studies in the last 20 years^3–5^. The original purpose of VOLCORE was to help understand the global picture of changing rates of large magnitude explosive volcanism through time, through comparison with other global volcanism datasets. The analysis of these data will be described in later papers. Secondary uses of the data are to assist in other kinds of study, for example the identification of tephra horizons which can then be sampled for geochemical analysis. VOLCORE does not include information on the identification (geochemically or otherwise) of tephra layers as specific volcanic eruptions; however this information can be found in some cases by a literature search.

**Fig. 1:**
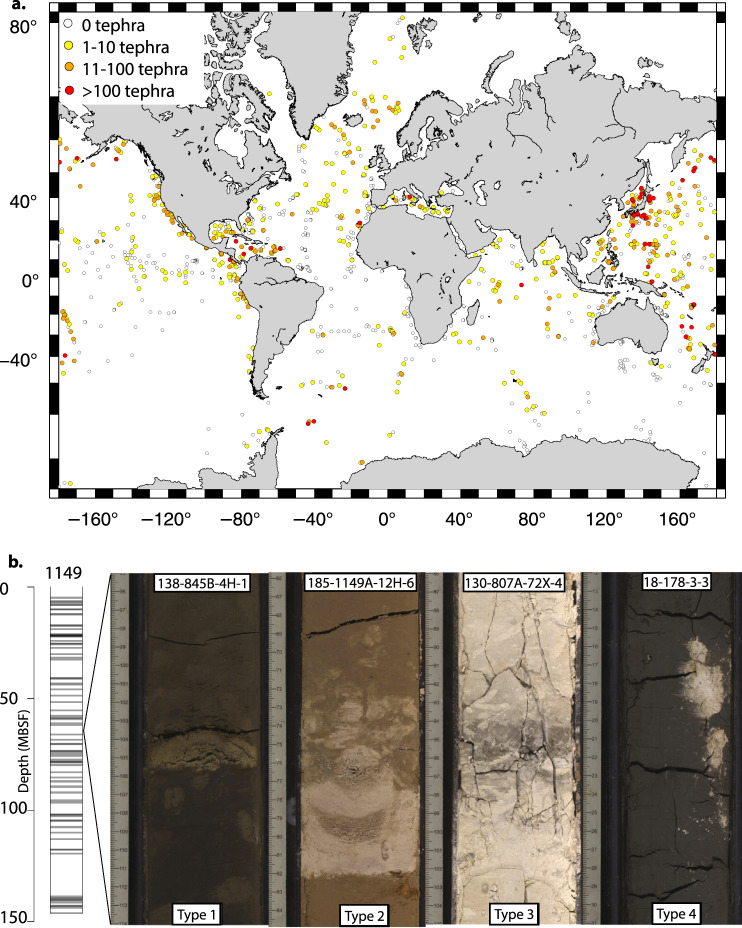
Spatial distribution of drill sites, example of the tephra record at one drill site and typical volcanic ash forms. (**a**) Map of the drill sites. The combined DSDP, ODP and IODP drill sites, each site may contain multiple drill holes within a small distance from one another (e.g. 50 m offset). Filled circles show locations with reported tephra, open circles are locations with no reported tephra. (**b**) Drill holes with tephra and volcanic ash forms. Each drill hole may have one or more tephra horizons, as shown by horizontal bars in the 1149A drill hole on the left of the diagram. If a series of tephra are dated, they form a time series of volcanic activity. The tephra horizons often take different forms, as shown by the volcanic ash occurrences on the right hand side of the diagram. The most common distinction is a layer versus a patch, however not all layers are the same. Type 1 layer has sharp upper and lower boundaries, Type 2 layer has a sharp lower, and bioturbated or gradational upper boundary, Type 3 layer has both upper and lower bioturbated/gradational boundaries, Type 4 is a patch or pod.

**Fig. 2:**
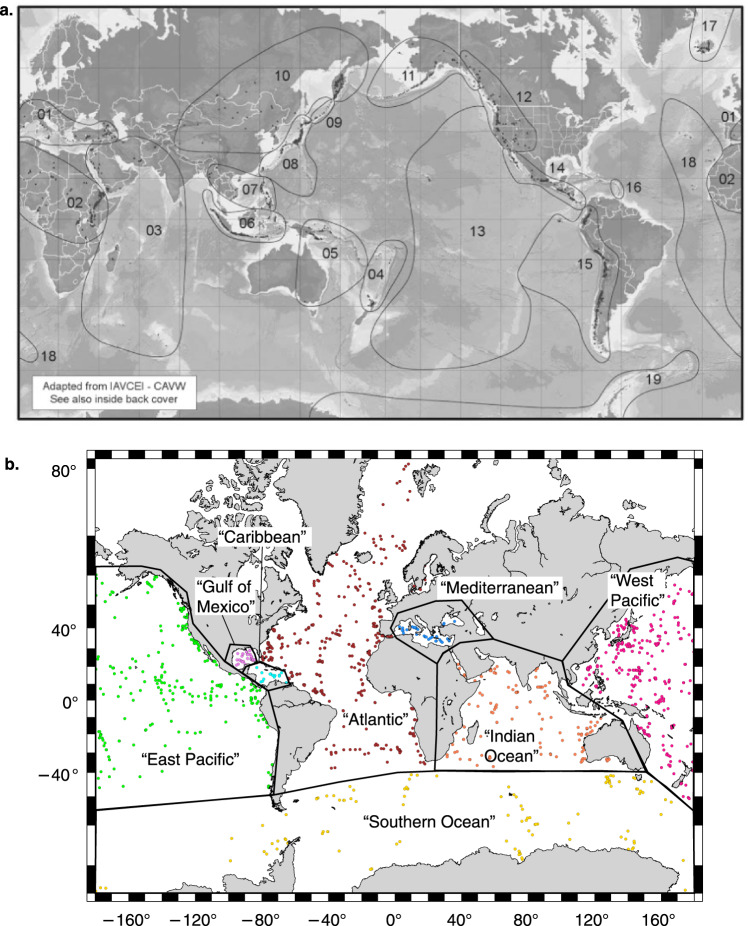
Maps depicting the spatial divisions used in the ‘Holes’ tab of the VOLCORE database. (**a)** shows the volcano regions used in the LaMEVE database, after Volcanoes of the World^13^. Dark triangles represent active volcanoes. (**b**) shows the drill hole locations (filled circles) divided into oceanic region. Region names given are those used in VOLCORE.

**Fig. 3:**
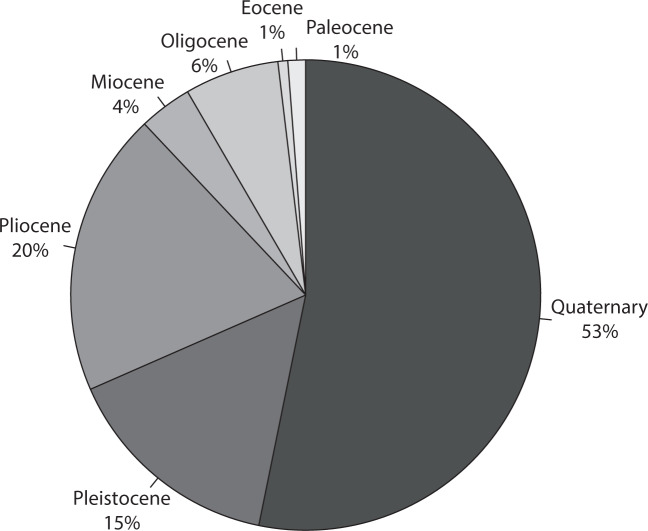
Pie chart showing the total number of tephra occurrences during geological time periods. The Quaternary dominates the VOLCORE record.

**Fig. 4:**
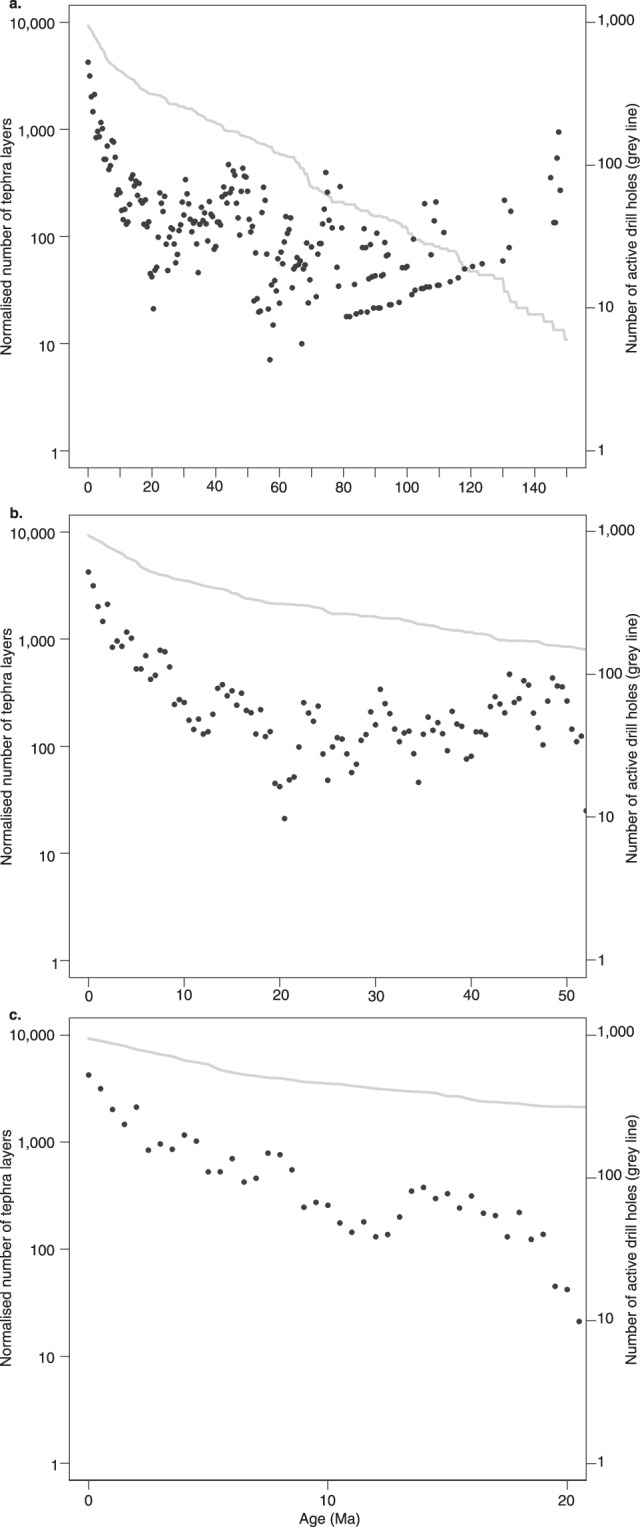
Number of tephra occurrences in 500 ka bins, normalised to the percentage of drill holes with material of that age. This normalisation removes the bias of there being more tephra recorded in recent times due to there being more core material from those times. Part A shows the whole record, back to 150 Ma, part B zooms in on the last 50 Ma and part C on the last 20 Ma. The tephra used in these plots are those with a content of ‘ash’, ‘ash-rich’, ‘ash-bearing’ and ‘ashy’. The tephra occurrences with ‘tuffaceous’ content (not shown here) create a peak at around 30 Ma that may represent erosion and reworking.

The OD programs recovered a total of 434,204 metres of core material during 285 Expeditions (data correct for expeditions with published Proceedings volumes until August 2019). Three main drill ships have been used, the Glomar Challenger for 96 DSDP expeditions, the JOIDES Resolution for 110 ODP expeditions as well as 56 IODP expeditions and the Chikyu for 15 IODP expeditions. Additionally, Mission-Specific Platforms (MSPs) have been used for 8 IODP expeditions where a ship outside the capabilities of the JOIDES Resolution or Chikyu was required, for example a specialist shallow water platform, or ice breaker ship. Figure 1 displays the spatial coverage of the drill holes that contain visible tephra, shows an example of an individual drill hole with tephra horizons, and illustrates examples of tephra in both layer and patch form. Table 1 gives some summary expedition, drilling and core recovery figures for the OD expeditions used in this study. A total of 34,696 tephra layers were collected, of which it was possible to estimate ages for 29,493 tephra layers. The 285 OD programmes expeditions drilled 3,886 holes, 1,154 of which reported tephra layers. Of those drill holes with tephra layers, 946 had age-depth models applied. Age-depth models are either generated during, or post-cruise. Notably 223 holes contain only 1 tephra layer. Table 2 demonstrates that nearly an order of magnitude larger numbers of drill holes contain between 1–10 tephra layers, than any other division (e.g. 11–20 tephra layers). Table 3 demonstrates the increase in the number of tephra layers recovered, from the 1960’s to recent time. Advances in drilling technology leading to improved core recovery and deeper drill holes may explain this increase. When the global data are divided into regions there are variations in numbers of tephra per drill hole (Table 4; Fig. 2), with the majority of tephra located in the West Pacific. The tephra ages listed in the database are dominantly during the Quaternary (Fig. 3). In order to get a preliminary overview of the data, and not over-count tephra layers due to intensive coring through certain time periods, we normalised the number of tephra layers by binning the data into 500 ka intervals, then dividing by the percentage of drill holes with material of that age (Fig. 4). The records of tephra (VCDs) in OD includes a diversity of descriptive terms used by onboard scientists. The most commonly used descriptive terms are listed in Table 5. Numbers of times that these words are used in VCDs to describe tephra are given in the table. Other information includes estimates of the percentage of times that tephra layers were confirmed or given as a false positive during ground truthing^6^. Ground truthing is the process of re-examining a sample of the original cores, to verify the accuracy of the original visual core descriptions. False positives represent original over-recording and false negatives represent original under-recording. Table 5 shows that the main descriptive term used by core describers falls under the term‘tephra’ (includes ash, tuff etc.) with much less use of other descriptors.

**Table 1:** Overview of DSDP, ODP and IODP statistics for expeditions used during data collection for the VOLCORE database.

	DSDP	ODP	IODP	IODP	Total
	(1968–1983)	(1985–2003)	(2003–2013)	(2013–2019)	
Expeditions completed	96	110	50	29	285
Sites visited	622	662	222	118	1,584
Holes drilled	1,116	1,791	641	338	3,886
Cores recovered	19,918	35,600	12,176	9,212	76,906
Core recovery (m)	97,334.30	221,318.40	66,635.88	48,915.46	434,204

**Table 2:** Number of tephra per drill hole in bins of 10 for up to 200 and bins of 100 for >200.

Number of tephra per drill hole	Number of drill holes with each number of tephra
0	2719
1–10	741
11–20	127
21–30	58
31–40	40
41–50	30
51–60	25
61–70	13
71–80	23
81–90	18
91–100	12
101–110	10
111–120	8
121–130	6
131–140	4
141–150	3
151–160	3
161–170	4
171–180	3
181–190	1
191–200	2
201–300	8
301–400	8
401–500	1
501–600	0
601–700	0
701–800	0
801–900	0
901–1000	3
>1000	3

**Table 3:** Number of tephra recovered by OD per year.

Year	Total tephra	Year	Total tephra
1968	45	1994	344
1969	206	1995	1110
1970	88	1996	2631
1971	353	1997	106
1972	282	1998	599
1973	307	1999	787
1974	151	2000	651
1975	29	2001	203
1976	50	2002	595
1977	509	2003	51
1978	1089	2004	2
1979	280	2005	18
1980	48	2006	0
1981	252	2007	148
1982	359	2008	160
1983	667	2009	1362
1984	0	2010	31
1985	172	2011	881
1986	226	2012	921
1987	1135	2013	1734
1988	416	2014	9212
1989	1661	2015	96
1990	700	2016	1800
1991	201	2017	405
1992	1112	2018	421
1993	90

**Table 4:** Regional variations in numbers of tephra recovered by OD.

	Atlantic	Caribbean	East Pacific	Gulf of Mexico	Indian Ocean	Mediterranean	Southern Ocean	West Pacific
No. of drill holes	1094	37	1002	62	364	116	250	961
No. holes with tephra	200	20	309	17	95	51	54	413
Total no. tephra	2,594	2,093	4,359	99	1,260	759	824	22,708
Max. tephra per hole	127	921	161	28	211	164	195	2,526
Mean tephra per hole	13	105	14	6	13	15	15	55
Median tephra per hole	3.5	15.5	5	2	5	3	3	9
Mode tephra per hole	1	3	1	2	1	1	1	2

**Table 5:** Summary of the most common terms in the ‘tephra form’ and ‘content’ columns in the ‘Layers’ tab of the database.

Content\Form	Layer	Patch	Mixed	Totals:	*False + (sub-sample)*	*Yes (sub-sample)*
Tephra	22,535	3,804	530	**26,869**	*20% (182)*	*80% (726)*
Tephra-rich	160	545	42	**747**	*52% (13)*	*48% (12)*
Tephra-bearing	4	0	5	**9**	*N/A*	*N/A*
Ashy	131	185	9	**325**	*29% (2)*	*71% (5)*
Tuffaceous	26	22	5,895	**5,943**	*N/A*	*N/A*
Vitric	17	3	8	**28**	*13% (22)*	*87% (141)*
Volcanic	46	21	62	**129**	13% (19)*	87% (124)*
Volcaniclastic	408	15	221	**644**	13% (19)*	87% (124)*
Totals:	23,327	4,595	6,772	34,696		

The physical form of each tephra is listed as either a layer, patch or mixed. This is decided by the data collection team by using the information given on the VCD. A patch can also be described as: pod, pocket, lens, bleb, blob, spot, speck, glob. The descriptions given in the VCD also give an indication of the volcanic content of the tephra occurrence. The left hand column lists the main volcanic descriptive terms that were used to identify tephra occurrences. These terms can cover several variations, for example here ‘Tephra’ includes ‘tuff’ and ‘ash’. The two right hand columns relate to the content descriptors that were checked during ground truthing^6^, note that only a subset of the VOLCORE data (1,246 of 34,696 tephra occurrences) were ground truthed. Relative percentages of False positives and confirmed tephras are given, with actual numbers in parentheses. ‘False + ’ indicates over-recorded tephra, ‘Yes’ indicates correctly identified tephra. False negative numbers are not included, as by definition they were tephra occurrences that had not been recorded in a VCD, and so have no descriptive terms. *‘Volcanic’ and ‘Volcaniclastic’ have the ground truthing numbers for the term ‘Volc’.

## Methods

### Definition of a tephra layer used in VOLCORE

The listing of a tephra layer in VOLCORE is based on descriptive criteria and does not imply any firm knowledge of source processes, i.e. primary fall, flow or reworking. The individual may glean some insights into sedimentary processes by examining the ‘tephra form’ and ‘content’ columns, where for example, ‘layer’ form combined with ‘tephra’ content would imply a seemingly more likely primary origin than ‘patch’ form combined with ‘volcaniclastic’ content. For each VOLCORE layer of interest, the user is advised to read the relevant OD reports volumes to make their own decisions about layer origin. In this report, general use of the term ‘tephra’ includes all grain size and lithification variations of the word (e.g. ash, tuff etc.). The less detailed nature of many older OD VCD records precludes consistent categorisation of records by grainsize in VOLCORE. The user can decide whether to use all of the data in VOLCORE, or a subset more suitable for their purposes. For example those OD sites located very close to volcanoes may be less likely to have exclusively tephra fall deposits. All OD sites with recorded visible tephra layers are included in VOLCORE, for completeness.

Tephra layer occurrences were defined and included in the dataset if a layer had >25% tephra content. This definition was decided upon to include as many tephra layers as possible. Layers with a lower percentage of tephra were thought likely to be reworked sediment. Data gathered in calibrations and testing study of Mahony et al.^6^ established that most (70%) tephra layers are almost entirely made of volcanic ash (see Fig. 3 in Mahony et al.^6^). Most of the tephra layers in VOLCORE reports are considered to be primary. This ‘ >25%’ tephra content definition largely worked well, with four noted exceptions of data collected from Expeditions 350, 351, 371 and 376. These expeditions were in an area with very high percentage of volcanic material had been mixed into the background muddy sediment, so what is essentially background sediment was often recorded as 25–75% volcanic grains and clasts (“tuffaceous” or “volcaniclastic”). These layers met our criteria, so are included. However, upon inspection of the cores, the primary tephras from these expeditions are those with >75% volcanic material, and the 25–75% are really layers related with deposition of mud rich in volcanic components. The data are included for completeness, but are marked as ‘tuffaceous’ or ‘volcaniclastic’. The user should note these exceptions and decide whether these data are useful for their study.

### Data source

Visible tephra layers are commonly conspicuous due to contrasts in colour, grain size and texture between the layer and surrounding sediments (Fig. 1). The boundaries between tephra layers and their surroundings can be sharp or diffuse; common varieties of volcanic ash layer (a tephra sub-type) contact are shown in Fig. 1b.

**Fig. 5:**
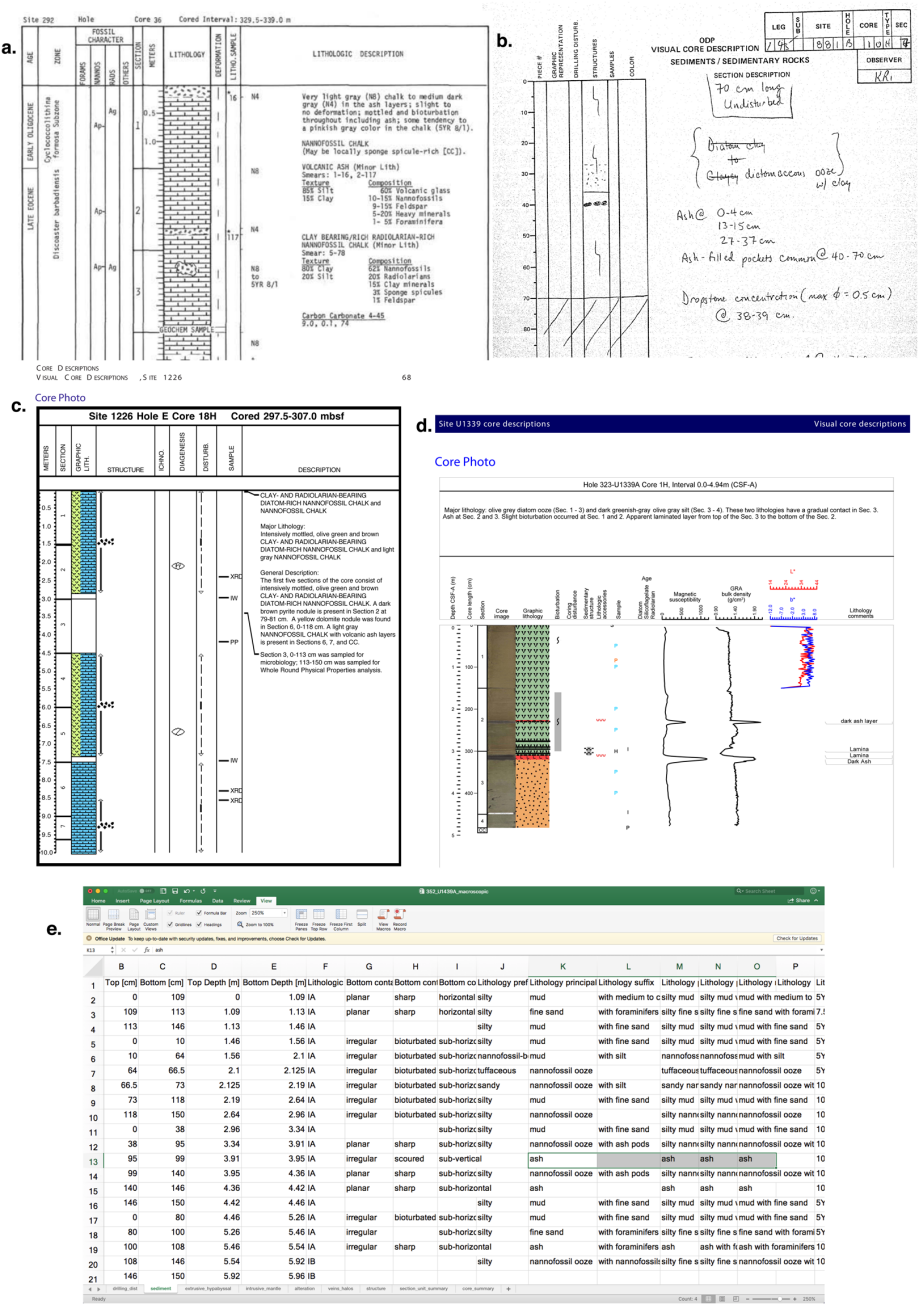
Examples of VCD types. (**a**) is a typed, and later scanned DSDP VCD. (**b**) is a handwritten ODP VCD. (**c**) is a digitised ODP VCD. (**d**) is a digitised IODP VCD. (**e**) is a fully digital IODP VCD data spreadsheet.

Tephra layers are recorded in the Visual Core Description (VCD) during systematic logging of sediment cores on board during an expedition. Every expedition produces reports which include the visual core description forms (VCDs), which were the primary source used for VOLCORE data collection. Each VCD is a visual log and description, of each 1.5 m long section of core material that is recovered to the ship. The VCD archive takes slightly different forms, through DSDP to recent IODP expeditions. During the DSDP the VCDs were typed up and published in hard copy format as part of the expedition reports. These VCDs have since been scanned and are available in pdf format, with 2 or more cores (each core up to 10 m long) on each A4 page (Fig. 5a). During the ODP either handwritten or digitised VCDs or sometimes both were published, with handwritten VCDs having one A4 page for each 1.5 m core section (Fig. 5b,c). IODP expeditions have changed from using both handwritten and digitsed formats, to using digital data capture, producing searchable VCD MS Excel files, where each cm of core is given a descriptive term (Fig. 5d,e). In most cases the original shipboard handwritten VCD forms are available, which were our primary source for data collection. If handwritten VCDs were not available, then digitised VCDs were used, or the text of the associated reports were searched for key terms (e.g. ‘tephra’, ‘ash’, ‘volc’, ‘tuff’, ‘glass’ etc.) to identify any tephra layers that were reported, but were missing from the VCD forms. All data were obtained free of charge from the IODP web pages.

### Data collection method

Visual core description forms for all OD expeditions were individually inspected for recording of tephra, ash, glass or other volcanic products. To be consistent across all of the expeditions only the shipboard VCDs were used as the data source, published post-cruise literature were not used. If the user is interested in a particular expedition, they should check the literature for post-expedition updates regarding tephra identification. VOLCORE provides a global overview of volcanism via a standardised data capture method, it does not include post-expedition studies. OD core description procedures and terminology are fairly standardised but have evolved with time. More importantly, definitions of the descriptive terminology used are listed in the lithostratigraphy methods section of reports associated with each expedition. Prefix and suffix qualifiers such as ‘with’, ‘bearing’, or ‘rich’ have specific meanings regarding the proportions of types of sediment observed, but can vary slightly between expeditions. Therefore it is generally straightforward to establish, for example, the proportions of a sediment referred to by the phrase “foram-rich ash layer with sponge spicules”. In this example, the main constituent is the ash, the next most common is the prefix (foram-rich), then the least common is the sponge spicules. Each of these will have a percentage range associated with them. Such descriptors were important for our data compilation, as early on we decided on set criteria for classifying a visible tephra occurrence. The criteria to include a tephra layer in the database are as follows:

1. Occurrence in sediments or lithified sediments only, no hard rock basalts etc. If an expedition cores (for example) both basalts and volcaniclastics, the volcaniclastics are recorded in VOLCORE, but the basalts are not.
2. Occurrences where defined descriptive qualifier (prefix and suffix) terms are used, the qualifier definitions must be within certain limits. The minimum threshold for a sediment to be considered volcanic in origin, was chosen as 25% tephra content. Any qualifier definitions were checked in the initial reports and methods to justify the inclusion of these sediments. The most commonly used word to describe tephra in a visual core description is the word ‘ash’, in more recent expeditions this has changed to the term ‘tephra’. Generally speaking, ‘with tephra’ and ‘tephra bearing’ were below the inclusion threshold as those qualifiers were assigned to sediment with varying tephra percentage, but almost always contained <25% tephra. The term ‘tephra-rich’ was usually included because it generally referred to sediment with >25% tephra content. ‘Tephra-rich’ or other commonly collected terms would not be included if the definition given in the methods (or explanatory notes) section of the expedition reports stated that the term was used to describe sediment where tephra contained less than 25% of tephra. This is the case for example in Expedition 31 (See section 1b of Explanatory notes: http://www.deepseadrilling.org/31/volume/dsdp31_01.pdf). In general, inclusion of the ‘ash-rich’ layers is justified by consideration of Mahony et al.^6^ ground truthing study which found that of those tephra occurrences described in VCDs as ‘ash-rich’, half were dominantly tephra. Sediments with <25% tephra were not considered as tephra, so not recorded in VOLCORE. Common examples of terms which were not recorded are: ‘volcanic sand’, ‘tephra bearing’, ‘… with tephra’, ‘ashy’, ‘siliceous’, ‘tuffite’, ‘tuffitic’. Tephra occurrences with greater than 25% ash were included. Common examples include: ‘ash/tephra rich’, ‘ash’, ‘sandy ash’, ‘tephra’, ‘V1’, ‘V’, ‘tuff’, ‘tephra with….’. Definitions of these terms can vary by expedition, so sometimes terms that are usually excluded, can be included; one example of this is the term ‘Ashy’.
3. Where qualifiers are not provided, e.g. in older DSDP expeditions, the basic descriptive terms are used and assessed on a case by case basis. For example, in DSDP Expedition 94, Hole 607A-11, a record is described as ‘ash-bearing’. Such a layer would normally not be included because ‘ash-bearing’ usually indicates a tephra content of less than 25%, but the associated smear slide is described as containing 60% volcanic glass, so it is included.
4. Representation of tephra horizons or patches on the graphic log, with or without a written description are included. A patch can also be described as a pod, pocket, lens, bleb, blob, spot, speck, glob.
5. Smear slide descriptions that include volcanic glass can be included as extra information if an additional description or graphical representation on the log is present. Smear slide descriptions were not included as a tephra occurrence if they were the only source of information, due to the non-continuous sampling spacing nature of smear slides. Smear slides are routinely used to identify tephra, however the slides are not always made permanent (and so not reported), sometimes time constraints mean that temporary (unreported) smear slides are used to identify tephra.
6. Descriptions of tephra including a question mark are included in the dataset but with the query notation, e.g. ‘ash?’ in the note column.
7. Layers described as ‘Altered ash’ were included.
8. Descriptions of individual pumice were not included.

Every drill hole with a VCD available was examined. VOLCORE does not summarise per drill site, each site may have multiple closely spaced (e.g. 50 m) drill holes, which may sample the same volcanic history. VOLCORE includes a site identifier field which allows the user to examine data by drill site where required. For each tephra layer that passed the criteria we extracted data on core location, on depth down the core and tephra layer thickness. We estimated the age of the layer using age-depth models for each drill hole. Age-depth models allow the conversion of a tephra depth below sea floor, to a tephra (or volcanic eruption) age. Where published age-depth models were available, they were used to estimate an age for each tephra layer. Shipboard age-depth models are typically generated from either magnetostratigraphy, biostratigraphy or both. Post-cruise improvements to age-depth models are common, for example by oxygen isotope analyses of foraminifera. In some cases the original age-depth tie point data were not available in tabular form. Ages and depths were picked off published plots and then used to estimate tephra layer ages. Where multiple age-depth models were available for a drill hole, they were occasionally combined, in order to extend or enhance the datable record. Here, the most up to date or complete age-depth models were applied to 946 tephra-bearing drill holes. Age-depth models were not found for 208 tephra-bearing drill holes, or models that covered the interval with the tephra layers. In each age-depth model the rate of deposition between tie points in the age-depth model is assumed to be linear, and any tephra layers outside of the depths used in the model were not assigned an age from that model. The source of each age-depth model is noted within the ‘AgeDepth’ tab of the dataset.

Summarising the age-depth data, the dating methods break down as follows with the numbers indicating how many age depth tie points are based on each method: biostratigraphy (10,006); magnetostratigraphy (3,995); oxygen isotopes (44,857); stratigraphy (4) and other (3). A further 921 tie points are either biostratigraphy, magnetostratigraphy or isotopes, but they are not individually distinguished. Isotope methods include application of isotope systems, such as δ^18^O, δ^13^C, Re-Os, and proxy methods, such as using the relationship between ^87^Sr/^86^Sr and seawater age to date carbonate components. The ‘other’ methods include a dated microtectite layer. Note that some age-depth models are based on more than one dating method, for example shipboard age-depth models are commonly based on a combination of magnetostratigraphy and biostratigraphy. Of the 946 age-depth models, 812 were found as tabular data, 134 were estimated from graphs from the initial reports volumes by the data collectors. The maximum number of age-depth points was 2521 in a model, whilst the minimum was 2, with a mean of 61.5 and a median of 11 tie points.

**Fig. 6:**
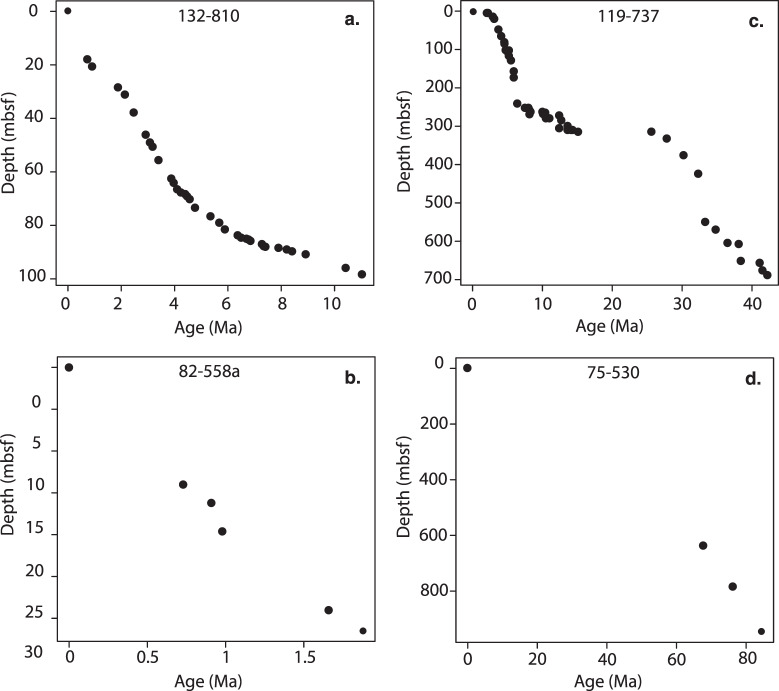
Age-depth model examples. Dots represent each age-depth tie point. These examples demonstrate that the number of age depth points is highly variable between models, and even within one age-depth model there is variation in the numbers of tie points with depth.

In VOLCORE we report the age estimates for the tephra layers without any in-depth assessment of the uncertainties for each age, which would require a significant effort to achieve. However, the user may find information provided in VOLCORE useful to estimate age uncertainties. Fields in the coring tab provide information regarding the dating method, how and where the data were obtained, whether they are primary data or an interpolation. The actual age-depth models that were used are provided, so that the user can assess the quality of the data source, as well as the number and spacing of tie points. In general, the main dating methods all have pros and cons. Oxygen isotope records provide high resolution age data and can be tied to the LR04 global data stack^7^ to provide high confidence ages. However, isotope data are generated post cruise and require significant laboratory work, so often only part (or none) of the cored interval is dated using this method. Biostratigraphy and magnetostratigraphy records are typically generated on board ship, so are more commonly available than isotope data. They are less precise than isotope data, but usually cover a much larger depth interval. Here we discuss a few examples of age-depth models to illustrate their varied quality. We also discuss uncertainties in the age estimates. The examples are shown in Fig. 6.

Expedition 132, Hole 810 is an example of an age depth model based on magnetostratigraphy (Fig. 6a). There are a large number of points (35) defined by boundaries between normal and reversed periods. For magnetic boundaries stratigraphy, the uncertainties in the absolute ages are typically of order 0.3 to 0.5 my for intervals >1 my^8^. This example with many calibration points, also allows some assessment of the linear extrapolation to estimate a tephra layer age between calibration points. Clearly over long periods of time the sedimentation rate is far from linear, but is for the most part reasonably linear over time periods comparable to the spacing of the age calibration tie points. Deviations from linearity over short time periods might represent real variations in sedimentation rate but they could also reflect uncertainties in the calibration of the geomagnetic time scale.

The age-depth model of expedition 82, Hole 558A (Fig. 6b) shows a more typical example with half a dozen points or so. There are fewer points defined by magnetostratigraphy but the data are also over a much shorter and more recent time period than Expedition 132, Hole 810. Here the chron ages are much more accurately dated. The Brunhes-Matuyama boundary is dated to 781 ky with a likely uncertainty of only a few thousand years. The data also suggest that the linear assumption is reasonably good and might lead to uncertainties in tephra layer ages that are less than 0.2 kyrs. This example illustrates that the quality of the age-depth model, from the point of view of estimating tephra layer ages, is a function of the quality of calibration point ages as well as the number of calibration points.

Expedition 119, Hole 737 is a good example of a well constrained age-depth model using biostratigraphy (Fig. 6c). There are plenty of tie points which are based on first and last occurrence biostratigraphy data.

The age depth model of expedition 75 Hole 530 (Fig. 6d) only includes four data points unevenly distributed in time. Thus tephra layers in between the youngest age at 67.75 my and zero will have very large uncertainties.

The user should bear in mind the criteria that were used to compile VOLCORE, and decide if the criteria used here fit the needs of their own work, before using the VOLCORE data. Exclusion of cryptotephra horizons is a feature of VOLCORE, primarily because it was deemed unfeasible for this project to thoroughly capture information regarding all cryptotephra horizons. VOLCORE attempts to capture visible tephra using consistent set criteria.

## Data Records

The data are available in .xlsx format and are stored in the PANGAEA online repository^9^.

The data were collected from 3,886 drill holes (expeditions 1–381), distributed across all major seas and oceans (Fig. 1). The data collected include information about the location, depth, thickness of the tephra, as well as any associated notes. The data are spread over five worksheets, one with details per tephra layer (or patch), one with details for each drill hole, one with the age-depth models used in this study, one with the coring information for each drill hole. A fifth sheet titled ‘Key’ describes the meaning of every column in the data worksheets. The individual data sheets can be linked via the common Hole and Site ID fields (HID, SID). Table 6 is an example of the collected layer data. The data are now described.

**Table 6:** Example of the collected data. These example data are from the ‘Layers’ tab. Note only a selection of columns are included in this example.

Exp	Hole	Core	Type	Section	Top_offset_cm	Age_Ma	VCD_notes	Tephra_form
145	887 C	27H	H	2	1.12	11.972	Ash-filled burrows	Patch
145	887 C	27H	H	5	1	12.6108	Ash-filled burrows	Patch
145	887 C	30H	H	6	0.94	17.9255	Ash layer, black, sharp base, grad top	Layer
145	887 C	30H	H	2	0.55	17.0523	Ash-filled burrows	Patch
146	888B	37X	X	7	0.23		Black ash layer? Pyrite?	Layer
146	888B	37X	X	7	0.28		Black ash layer? Pyrite?	Layer
149	897D	6 R	R	1	0.3		Altered tuff	Layer
151	907 A	2H	H	3	0.91	0.54646	Silty ash	Layer
151	907 A	2H	H	4	0.91	0.61561	Possible ash layer	Layer
151	907 A	2H	H	4	1.27	0.63302	Possible ash layer	Layer
151	907 A	2H	H	4	1.33	0.63786	Clasts of ashy material	Patch
151	907 A	2H	H	6	1.3	0.78342	Ash layer/sandy	Layer

**Layers tab –**

Each row corresponds to a specific tephra layer.

**HID** (column A). Unique identifying number for each Hole.

**SID** (column B). Unique identifying number for each Site (a Site often includes multiple closely spaced Holes).

**LID** (column C). Layer identification number, each row is numbered sequentially.

**Exp** (column D). DSDP, ODP or IODP expedition number.

**Hole** (column E). This is the official drill site and hole designation; for example site 1149, hole A is given as 1149A. If a letter precedes the Hole number this refers to the implementing organisation drill ship, ECORD Mission specific platform (M), USIO JOIDES Resolution (U) or the Japanese JAMSTEC Chikyu (C). Some expeditions have a name rather than a number, e.g. JFAST3. There are commonly several drill holes per site, the drill holes will be located close to one another (e.g. 50 m), to attempt to sample the same sedimentary history at each hole. Drill holes either sample the same sediment intervals (e.g. 0–50 mbsf) or have overlapping intervals to extend the record deeper (e.g. hole A: 0–50 mbsf, hole B: 40–90 mbsf). A new drill hole may be started if the previous drill hole reaches an interval that is difficult to core, maybe due to changes in sediment lithification. In that case a different drilling technique may be required, e.g. the change from using advanced piston coring (APC) to extended core barrel (XCB) and rotary core barrel (RCB) drilling. Different drilling techniques require different diameter drill holes, hence the need for a new hole upon the change in coring technique. Twenty two holes have been drilled more than once, because a later expedition re-visited the original drill hole (Table 7). Each hole consists of multiple cores.

**Table 7:** Holes that are duplicated in the dataset, due to later expeditions re-visiting a drill hole.

Hole	Expeditions which visited the Hole
395 A	45/78/336
417D	51/52
418 A	52/53
504B	69/70/83/92/111/137/140/148
603D	93/95
603E	93/95
603 F	93/95
648B	106/109
735B	118 / 176
801 C	129/185
856H	139/169
857D	139/169
858 G	139/169/341 S
1256D	206/309/312/335
C0010A	319/332/365
C0002G	332/338
U1309A	304/340 T
U1309B	304/340 T
U1309C	304/340 T
U1309D	304/305/340 T
U1309E	304/340 T
U1473A	360/362 T

**Core** (column F). This is the core number, assigned during drilling. The core numbers increase incrementally from the surface downwards. Core barrels are 10 m long, but core recovery can vary from an empty core barrel to > 10 m of sediment recovery, due to expansion. When there is partial recovery it is not known exactly where within the 10 m interval the material originated, so the sediment in the core barrel is pushed along to the top end of the barrel. Coring can be continuous, e.g. core 1: 0–10 m, core 2: 10–20 m etc. or specific intervals can be targeted where the drillers ‘wash’ down to a specific depth before coring begins. Continuous coring may not always be an exact record of the sediments due to material falling into the drill hole or other coring disturbances (see Jutzeler et al.^10^).

**Type** (column G). The letter after the core number represents the type of drilling, as described in the ODP Core Lab Cookbook^11^ as well as in the operations summary of expedition reports: RAB-C (A), Bit Sample (B), HYACE Rotary (E), Half-length Advanced Piston Core (F), Advanced Piston Core (H), Baker Hughes INTEQ coring system (L), Navi-Drill Core Barrel (N), Rotary Core Barrel (R), Extended Punch Coring System (T), Wash Core Sample (W) and Extended Core Barrel (X). Each core consists of one or more sections.

**Section** (column H). This is the section number, assigned during curation. Section numbers increase incrementally, starting with section 1 at the top of the core. Cores are cut into 1.5 m sections. Often the last (deepest) section in a core is not a full 1.5 m in length. There is commonly a core catcher section from the very bottom of the core, this is designated ‘CC’ and is usually around 20 cm in length.

**Top_offset_m** (column I). The top of the tephra layer in metres from the top of the section, given to the nearest centimetre. This is generally a number between 0–1.5. However, some tephra layers were noted as a continuation from the previous core. In this case the tephra is given the section number of the lowermost section (the base of the tephra layer), and the top of the tephra layer is given a negative value. For example, where the top 5 cm of the tephra layer is located in the previous section, then the top value is noted as “−0.05”. This case was only applied where a tephra layer is spread over one or more sections, but was not applied to one or more cores due to the possibility of an inexact match between consecutive cores. Where only one depth value of a tephra layer was given, and no thickness value, this depth value was put in both the top and bottom columns. If a tephra layer is recorded e.g. “there is a 2 cm ash layer” but there is no mention of at what depth the layer is, then the depth is recorded as the top of the section. If the tephra occurrence is described as a patch and no depth or thickness measurements are given, but it is marked on the VCD using the symbol appropriate to that expedition, then the depth of the patch was estimated from the log.

**Bottom_offset_m** (column J). The bottom of the tephra layer interval in metres, to the nearest centimetre, from the top of the section that it is located in. This is generally a number between 0–1.5. However, some tephra layers were noted as a continuation from the previous core (see ‘Top’ section for more details). Where only one depth value of a tephra was given, and no thickness value, this depth value was put in both the top and bottom columns. Where a depth and thickness were given, the depth goes in the bottom depth column, and the thickness in the thickness column. If a tephra layer is mentioned e.g. “there is a 2 cm ash layer” but there is no mention of at what depth the layer is, then the depth is put as the top of the section. If the tephra is described as a patch and no depth or thickness measurements are given but it is marked on the VCD using the symbol appropriate to that expedition, then the depth of the patch was estimated from the log.

**Depth_of_section_top_m** (column K). The top of the section in which the tephra layer occurs, in metres below sea floor, as recorded during core curation.

**Depth_of_layer_top_m** (column L). The depth below sea floor to the top boundary of the tephra layer, in metres. This is calculated using columns I and K.

**Depth_of_layer_bottom_m** (column M). The depth below sea floor to the bottom boundary of the tephra layer, in metres. This is calculated using columns J and K.

**Thickness_m** (column N). The thickness of a tephra layer, given in metres. The thickness may be reported in the core description on board ship, in which case the value was transferred directly to the datasheet. Where a tephra layer was noted, but either top and/or bottom or thickness measurements were omitted in the core description, then the data collector estimated the thickness from the visual log. Where there was no thickness given, or no way to estimate the thickness, the thickness was entered as ‘0’. The thickness generally ranges from 0–1.5 m. However, some thick tephra layers occur over multiple consecutive sections. If the tephra is described as a patch and no depth or thickness measurements are given but it is marked on the VCD using the symbol appropriate to that expedition, then the vertical dimension of the patch was estimated from the log. Note that a measurement given for thickness of a patch is less likely (than the thickness of a layer) to be the original deposit thickness, and so should not be used to compare with other tephra thicknesses.

**Thickness_depth_source** (column O). This column contains either a ‘Value_given’ or an ‘Estimated’ value, to represent whether the thickness value is given in the VCD or is estimated by the data collector from the VCD log. This designation was determined by the data collection team. These data can be used to identify records that have a less uncertain thickness value (Value_given). When these ‘V/E’ data are used in combination with the ‘Tephra form’ data (column R), the most reliable thickness data would be those that are classified as both ‘Value_given’ and ‘Layer’.

**Age_Ma** (column P). This field is populated by the tephra layer age, estimated by applying a linear age-depth model to the depths of the base of each tephra layer in mbsf (column M). The most recent, or most complete (for the intervals with tephra layers) age-depth models were chosen by the data collection team. However, age-depth models vary in quality (see earlier discussion). Information is recorded in the ‘Holes’ and ‘AgeDepth’ tabs detailing the source of each age-depth model, number of age-depth tie points, type of dating method used (e.g. magnetostratigraphy, biostratigraphy, oxygen isotopes) and the oldest age-depth tie point.

**VCD_notes** (column Q). Any notes that were written by the shipboard core describers on the VCD are in this column.

**Tephra_form** (column R). The physical form of the tephra occurrence. ‘Layer’ form is a band across the whole width of the core (see Fig. 1b). ‘Patch’ is a deposit that only partially crosses the core. ‘Mixed’ form includes deposits that are banded, interbedded, massive, laminated and other mixed. Categorisation of tephra form is by these authors from observing the VCD, not categorised during the initial shipboard studies.

**Content** (column S). Broad divisions using VCD tephra descriptors to indicate tephra content for each occurrence. Terms include: Tephra (this includes ash and tuff), Tephra-rich, Tephra-bearing, Sand, Siliceous, Tuffaceous (e.g. tuffaceous mud), Vitric (e.g. vitric silt), Volcanic (e.g. volcanic sand) and Volcaniclastic (e.g. volcaniclastic mudstone).

**Layer_comments_by_these_authors** (column T). Any additional notes made by the data collection team during data collection, were added here.

**LR04_Age_Ma** (column U). This column records the age of any tephra located in a drill hole which was included in the original LR04 stack by Lisiecki and Raymo^7^ and has ages calculated using the LR04 high resolution oxygen isotope data.

**Holes tab –**

**HID** (column A). Hole identification number, each row is numbered sequentially.

**SID** (column B). Unique identifying number for each Site (a Site often includes multiple Holes).

**Exp** (column C). Expedition number, as assigned by OD. Some expedition numbers are followed by a letter, indicating that it is not a main expedition. An ‘S’ indicates that it is an engineering expedition. A ‘T’ indicates that the ship is in transit between expeditions with a stop en-route for a short science operation.

**Hole** (column D). Hole code, as described in ‘Layers tab’ section.

**Latitude_dec_deg** (column E). Latitude of the drill hole in decimal degrees, taken from those given in the IODP Google Earth Holes .kml file overlay.

**Longitude_dec_deg** (column F). Longitude of the drill hole in decimal degrees, taken from those given in the IODP Google Earth Holes .kml file overlay.

**Coring_year** (column G). Year that the offshore drilling part of the expedition ended. Dates taken from drilling reports.

**LaMEVE_volcanic_region** (column H). The LaMEVE database is a land-derived record of Quaternary explosive volcanism^12^. If the drill hole lies within one of the ‘volcano regions’ defined for LaMEVE as outlined in Fig. 2a, then this column notes the volcano region number, which enables comparison with the volcanic history of that region. These divisions are added by the data collection team.

**Ocean_region** (column I). Similar to ‘LaMEVE’ in column F, this column is a spatial division of the data. Broad spatial divisions decided by the data collection team (Fig. 2b) divide the data into ocean regions.

**Latitude_zone** (column J). Location of the drill hole, in latitude zones: Arctic_circle (90°−66.55°N), N_hem (66.55°N-23.43674°N), Tropical (23.43674°N-23.43674°S), S_hem (23.43674°S-66.55°S), Antarctic_circle (66.55°S-90°).

**Total_no_cores** (column K). Total number of cores curated at this drill hole.

**Total_n_tephra** (column L). Number of tephra layers in this drill hole.

**N_dated_tephra** (column M). Number of dated tephra layers in this drill hole.

**N_tephra_no_age** (column N). The number of tephra layers in this drill hole with no age data. Either there is no age-depth model available for this drill hole, or the tephra layers occur deeper than the deepest point in the age-depth model that is available.

**Oldest_age_depth_point_Ma** (column O). Oldest age-depth point in Ma used in the age-depth model that was applied to the tephra layer data in each drill hole. Any tephra layer that is deeper than the depth related to the oldest age-depth point, do not have the age-depth model applied to them.

**N_age_depth_points** (column P). Number of age-depth points in the age-depth model that was applied to the data for each drill hole.

**Data_source** (column Q). Which type of VCD were checked, e.g. HVCD (hand written VCD), DVCD (digitised/scanned VCD), digital data (spreadsheet), or report text.

**Hole_comments_by_these_authors** (column R). Additional notes from the data collection team, to help understand inclusion or exclusion of ambiguous cases. Where a Hole or core is mentioned then that comment is directed to that. Where the comment appears on each Hole for an expedition, the comment is more general, or the describer did not note the exact Hole or core to which it is related.

**Age-depth models tab -**

The age-depth models that have been used are documented in the Age-Depth tab of the datasheet. Information includes each pair of age-depth tie points. Each row is one age-depth tie point:

**HID** (column A). Unique identifying number for each Hole.

**SID** (column B). Unique identifying number for each Site (a Site often includes multiple Holes).

**ADID** (column C). Age-depth tie point identification number, each row is numbered sequentially.

**Exp** (column D) See ‘Holes’ tab description.

**Hole** (column E) See ‘Holes’ tab description.

**Age_Ma** (column F) The age of the tie point, in millions of years (Ma).

**Depth_m** (column G) The depth of the tie point, in metres below sea floor (mbsf).

**Reference** (column H). Source reference(s) for the data that make up this age-depth tie-point, and model. References to initial reports or proceedings reports often have a direct URL link to the original data table at the end of the reference.

**Estimated_vs_Data_given** (column I). Whether the age-depth data tie points are estimated (E) (digitised off a graph or estimated from age-profile/text information), or whether the data were originally given in numerical format (D).

**Combo** (column J). Whether the age-depth data come from one source (N) or are combined from more than one source (Y) by the VOLCORE team.

**AllHole** (column K). Where the same age-depth model is used for all drill holes at a single drill site (Y). Where drill holes at a single site use different age-depth models (N). Where there is only one drill hole at a site with dated tephra (NA).

**DataType** (column L). If these authors regard the data as primary data points, they are marked as “Raw”. If these authors regard the raw data as already interpolated, they are marked “Interp”.

**AD_Type** (column M). Whether this tie point was based on magnetostratigraphy (Mag), biostratigraphy (Bio), Isotopes (Iso), or other methods (Other, or named method).

**AD_comments_by_these_authors** (column N). Any additional notes made by these authors were added here, for example distinguishing the type of isotope measurements used.

**Coring tab -**

The coring tab provides information to guide the data user to understand if they have a full or partial sediment record for each drill hole.

**HID** (column A). Unique identifying number for each Hole.

**SID** (column B). Unique identifying number for each Site (a Site often includes multiple Holes).

**CID** (column C). Coring data identification number, each row is numbered sequentially.

**Exp** (column D). See ‘Holes’ tab description.

**Hole** (column E). See ‘Holes’ tab description.

**Core** (column F). See ‘Layers’ tab description.

**Type** (column G). See ‘Layers’ tab description. Also, numerical value indicates no core recovery. Some early expeditions show multiple cores with the same identifier name, but different coring type.

**Top_depth_cored_mbsf** (column H). Top depth cored. Where there is an option, CSF-A depth scale is recorded.

**Bottom_depth_recovered_mbsf** (column I). Bottom depth recovered. Where there is an option, CSF-A depth scale is recorded.

**Key tab -**

**Name** (column A). A list of the column headers in the Layers, Holes, Age-Depth and Coring tabs.

**Description** (column B). Descriptions of the column headers in the Layers, Holes, Age-Depth and Coring tabs.

**Allowable_values** (column C). Types of data (numerical or alphanumeric) in each column in the Layers, Holes, Age-Depth and Coring tabs.

**Missing_data** (column D). Whether data may be missing in each column in the Layers, Holes, Age-Depth and Coring tabs.

## Technical Validation

Early in the database development the reliability of the shipboard VCDs was studied^6^. 1,241 tephra occurrences were ground truthed, by examining the physical cores that are stored in the IODP repositories, to test for the presence of tephra. A variety of tephra were examined (thick, thin, deep, shallow, recently cored, not recently cored etc.) and, although some differences were observed between expeditions, approximately 70–75% of tephra were correctly identified in the shipboard VCD forms (Table 5). Over-recording (false positives) and under- recording (false negatives) of tephra layers occurred on average 19.2% and 10.5% of the time, respectively, suggesting a slight tendency towards over-recording tephra layers. This result indicates that IODP training procedures are typically thorough, as each expedition has different science teams on board producing the VCDs, with different skills and interests. The general consistency of these results gave confidence in the use of the VCD record as a reliable data source.

The data collection process to form the VOLCORE database involved 4 data collectors reading through the shipboard VCDs and recording the appropriate information. Each collector followed the data collection guidelines given above, and if there was any doubt then the protocol was to discuss ambiguous cases with at least one other member of the team. The data collection team calibrated themselves using test cases in the beginning, and at least monthly afterwards.

## Usage Notes

While the purpose of this paper is not to analyse and interpret the data in detail, we mention some of the issues with the data that will need to be considered for analysis and interpretation.

### Issues with the data collection and verification

Tephra layers were identified in the VCDs by four people, so minor variations in applying the described collection criteria can be anticipated. The criteria were developed to try to ensure each data collector included the same tephra layers as another. In the case of ambiguity, consultation with another team member on the data collection team led to a joint decision for inclusion of a tephra or not, based on the criteria listed. Regular discussion between team members ensured everyone was calibrated to make it likely that the criteria were applied consistently, and judgements were the same. Ambiguous cases are summarised by expedition, in notes within the database explaining why certain tephra layers either were or were not included.

### Issues with the tephra layer record reliability

A concern in developing the database was that it may be unreliable due to the differences in core description detail between varying teams of core describers over several decades. As discussed in the technical validation section, the general consistency of the ground truthing results^6^ gave confidence in the use of the VCD record as a reliable data source.

### Thickness of a layer

Depositional and erosional processes may well have modified the thicknesses and location of tephra and consequently affected preservation. Tephra layers on bathymetric highs can be subject to current erosion for example leading to layer thinning or removal. In bathymetric lows re-deposition due to current activity or slumping can thicken layers and redistribute tephra away from the site of original deposition. Bioturbation levels can vary between sites, resulting in variations related to differences of mixing tephra into overlying sediment and thereby reducing layer thickness or number. There is wide variation in the structure of tephra layers (Fig. 1b) which affects how easily a meaningful thickness can be measured by the shipboard core description team. In some cases the layer has a well-defined sharp upper and lower contact with surrounding sediment (Type 1 in Fig. 1b). In such cases the uncertainty in thickness data can be attributed to two main factors: the physical measurement of the observed layer, and whether the deposition, preservation and coring processes preserve a layer with a reliable representative thickness. The physical measurement of a layer is usually taken to the nearest centimetre or half centimetre. However, many tephra layers do not form a neat layer with distinct top and bottom contacts, thus measuring the thickness reliably can be problematic for the describer. Commonly a layer will be sloping, disturbed (by sedimentary processes or coring), or have diffuse rather than sharp upper or lower boundaries (Fig. 1b). This can lead to over or under estimations of layer thickness. Bioturbation can lead to mixing of background sediments into the layer, which either thickens, diffuses, or removes the layer from the visible record. Coring processes can disturb the original thickness of a layer, especially if the layer is thick and coarse, or if it is located at the join between two cores. The tephra thickness data that were recorded in this dataset are either the values listed on the VCD or are estimated off a visual log. As such, where the thickness values are already given in the VCD, any uncertainties relating to measuring the actual thickness occur during core description. Where the thickness is estimated off of the visual log, there is uncertainty from both how the log is drawn, as well as read and recorded by the data collection team. Those tephra records where the thickness data are ‘given’ (not ‘estimated’), where the form is a layer, and the content is that with greater amounts of tephra (e.g. ‘tephra’ or ‘tephra-rich’) could be considered to be the most reliable for thickness studies. Values of thickness given for patches should not be considered as a true thickness measurement that can be used to compare with other tephra occurrences, but instead simply as the vertical dimension of the patch.

### Presence or absence

The presence of a tephra layer in cores can vary within a very small geographical area. The reasons that affect layer thickness (bioturbation, depositional and coring processes) can also affect whether or not a visible layer is preserved in a core. A thin tephra layer in one core may be represented by dispersed tephra (cryptotephra) in another core. Cryptotephra are not reported in VOLCORE, but form an important part of the tephra record. If a tephra layer is preserved and cored successfully, it then needs to be identified and reported in the VCD. Two teams of core describers operate onboard ship, where identification and interpretation of tephra will naturally vary to some degree; for example one team may report a small ash patch, that another team may not deem significant enough to report.

### Consistency between expeditions

Each expedition has different aims, with different core describers and levels of experience of tephra identification and description. Some expeditions core significantly more material than others, so time can be very short, thus for example <1 cm thick tephra layers may not be reported in one expedition, but would be in another. Colour changes due to oxidation of the core material can affect whether tephra layers are visible, so the time of description may influence the reliability of the VCD. Different data presentation formats (e.g. handwritten VCDs vs. digitised, vs. digital data capture) affect the record by the resolution of the core material presented. Large numbers of tephra layers may lead to thinner layers or patches being omitted, whereas if tephra layers are scarce, even a very minor patch may be reported. Mahony *et al*.^6^, investigated these issues and concluded that these factors overall result in minor differences.

### Tephra ages listed in VOLCORE

The ages of tephra horizons listed in VOLCORE should be regarded as estimates. The age-depth models applied by these authors attempt to encompass the most up to date age-depth models available, however it is possible that better age-depth models were missed during our searches. As described in the earlier section, there is much information available in VOLCORE to help the user begin to understand the range of uncertainty surrounding each age-depth point, but we do not explore these uncertainties here.

### Caveats on data interpretation

Our purpose in this report is to collate the data on occurrences of tephra layers in ocean drilling cores with minimal interpretations. However, we make some comments above on issues that need to be born in mind.

Other factors not detailed here may affect the record. For example, where whole round cores are sampled prior to description for interstitial water analysis, then tephra occurrences within these sections will be not be reported.

As a record of volcanism on a regional scale any single core might be missing events due to factors like dispersal direction. A collection of sites in a region will have duplication of the same tephra layers in different cores. The user should filter the data according to their own needs, for example choosing sites which are greater than a specified distance from a volcano if their aim is to try to only observe distal deposits from large magnitude eruptions. Lists of volcano locations can be found elsewhere (for example the Smithsonian website^13^), but due to the large temporal range of volcanic deposits covered by VOLCORE (0–150 Ma), no volcano locations are included within VOLCORE because comprehensive volcano location lists are unavailable for this long duration.

An important question to answer is whether VOLCORE represents the ‘true’ picture of volcanic deposits at any given drill hole, or if it is a set of random data. This is an issue all volcanic record compilations have to deal with, to try to understand how close the recorded data are to informing us of the true frequency or magnitude of volcanic events through space and time. All volcanic records are flawed in some way, whether it be reduction in detail as the record goes further back in time, or due to natural processes such as erosion, making it impossible to perfectly re-create previous event records. Statistical interrogation of VOLCORE and comparison with other global volcanic records can help to indicate whether VOLCORE captures true variations in the volcanic record. The creation of VOLCORE followed detailed data collection criteria, giving a transparent basis to try to determine the true nature of the data. Our initial hypothesis is that each tephra record in a single drill hole in VOLCORE represents a separate volcanic event, but this may not be true for all layers. The question of data robustness cannot be fully answered in this data descriptor, but is a topic for discussion in future publications.

### Future work

The database will need to be updated and we encourage PI’s of future IODP expeditions to devote time to populating it with newly discovered tephra layers. While checking the entire database by relogging core would be ideal it is very unlikely that resources could be found for such a herculean task. However, from time to time cores will be relogged for new research and here there is an opportunity for the researchers to correct any errors, add in new tephra layers and update the ages if new and improved age-depth models are developed. Future additions to VOLCORE could include fields containing information on tephra that are identified as specific eruptions or from certain volcanoes, new tephra layers identified during post-cruise work, the inclusion of identified cryptotephra horizons and the addition of geochemical data. There is scope for better characterisation in the uncertainties in the ages.

## Data Availability

No custom code was used in the development of VOLCORE.
